# The methods and outcomes of cultural adaptations of psychological treatments for depressive disorders: a systematic review

**DOI:** 10.1017/S0033291713001785

**Published:** 2013-07-19

**Authors:** N. Chowdhary, A. T. Jotheeswaran, A. Nadkarni, S. D. Hollon, M. King, M. J. D. Jordans, A. Rahman, H. Verdeli, R. Araya, V. Patel

**Affiliations:** 1 Centre for Global Mental Health, London School of Hygiene and Tropical Medicine, UK, and Sangath, India; 2 Institute of Psychiatry, King's College, London, UK, Public Health Foundation of India, and Sangath, India; 3 London School of Hygiene and Tropical Medicine, UK; 4 Vanderbilt University, Nashville, TN, USA; 5 Faculty of Brain Sciences, University College London Medical School, UK; 6 HealthNet TPO, Amsterdam, The Netherlands; 7 University of Liverpool, UK; 8 Teachers College, Columbia University and Columbia College of Physicians and Surgeons, NY, USA; 9 University of Bristol, UK

**Keywords:** Cultural adaptation, depression, developing countries, ethnic minorities, psychological treatment

## Abstract

**Background:**

Cultural adaptations of evidence-based psychological treatments (PTs) are important to enhance their universal applicability. The aim of this study was to review systematically the literature on adaptations of PTs for depressive disorders for ethnic minorities in Western countries and for any population in non-Western countries to describe the process, extent and nature of the adaptations and the effectiveness of the adapted treatments.

**Method:**

Controlled trials were identified using database searches, key informants, previous reviews and reference lists. Data on the process and details of the adaptations were analyzed using qualitative methods and meta-analysis was used to assess treatment effectiveness.

**Results:**

Twenty studies were included in this review, of which 16 were included in the meta-analysis. The process of adaptation was reported in two-thirds of the studies. Most adaptations were found in the dimensions of language, context and therapist delivering the treatment. The meta-analysis revealed a statistically significant benefit in favor of the adapted treatment [standardized mean difference (SMD) −0.72, 95% confidence interval (CI) −0.94 to −0.49].

**Conclusions:**

Cultural adaptations of PTs follow a systematic procedure and lead primarily to adaptations in the implementation of the treatments rather than their content. Such PTs are effective in the treatment of depressive disorders in populations other than those for whom they were originally developed.

## Introduction

Although there is extensive evidence of the effectiveness of psychological treatments (PTs) for depressive disorders (Cuijpers *et al.* 2008; Hollon & Ponniah, 2010), it has been argued that PTs are developed in particular cultural contexts and that this may limit their universal applicability. Adapting evidence-based PTs to incorporate elements that are contextually relevant and meaningful in the culture in which they are being delivered is recognized as an important step to increasing acceptability of the treatment, patient satisfaction and, ultimately, their effectiveness (Bernal & Scharrón-del-Río, 2001; Sue, 2003; Castro *et al.* 2010). Cultural adaptations of PTs can be viewed as a middle ground between the two extreme positions of considering an original evidence-based intervention as applicable to all cultural groups without the need for adaptation and a culture-specific approach with emphasis on unique culturally grounded content and process. This middle ground ensures that a cultural adaptation attempts to maintain fidelity to the core elements of the PT while adding certain cultural elements to enhance its acceptability and effectiveness (Falicov, 2009; Barrera *et al.* 2013). Understanding the process by which researchers have made these adaptations and the specific nature of the cultural adaptations may serve to inform others interested in tailoring PTs to specific populations of diverse cultures.

Although there have been previous systematic reviews of adaptations of mental health interventions (Griner & Smith, 2006; Huey & Polo, 2008; Benish *et al.* 2011; Smith *et al*. 2011), these have been heterogeneous in their scope (for example, addressing multiple mental health problems) and have left undescribed the process or nature of the adaptations. Notably, although there is a growing body of evidence supporting the effectiveness of adapted PTs for depressive disorder in non-Western countries, many of which have been adapted to the local context (Bolton *et al.* 2003; Rahman *et al.* 2008; Patel *et al.* 2011), these have not been included in existing reviews. The aims of this review were to synthesize the literature on cultural adaptations of PT for depressive disorder for both ethnic minorities in Western countries and culturally diverse populations in non-Western countries in three respects: (1) to describe the procedures used to adapt the PT; (2) to describe the extent and nature of the adaptations to the PT; and (3) to assess the effectiveness of the adapted PT.

## Method

We used standard methods for systematic reviews and meta-analyses in accordance with the PRISMA (Preferred Reporting Items for Systematic Reviews and Meta-Analyses) statement (Moher *et al.* 2009).

### Identification of studies

Studies were identified by a systematic literature search using the following strategies:

(1) A database search of Ovid Medline, EMBASE and PsycINFO until December 2011 was conducted to identify controlled trials conducted in Western countries with ethnic minority populations. The search terms used are described in Appendix 1 in the online Supplementary material. For studies from non-Western countries, the terms for ethnic minority populations were replaced with individual country names (i.e. World Bank-defined low- and middle-income countries as these would ensure inclusion of mainly non-Western countries). No start date was specified.
(2) Cross-referencing of eligible articles to identify additional studies that met our inclusion criteria.
(3) Key informants (i.e. known PT experts, including authors of the eligible studies) were contacted to identify other studies that could be included in our review.
(4) Bibliographies of key reviews of PTs (Patel *et al.* 2009) were hand searched to identify studies that may have been missed through the database search.

### Inclusion criteria

(1) Randomized controlled trials (RCTs) or non-RCTs that described the evaluation of PTs for depressive disorder in an ethnic minority population in a Western country or any evaluation of a PT in a non-Western country. Non-RCTs were included as they too would provide information on all the research questions including effectiveness.
(2) No restriction on language, sample size, type of comparison group or outcome measure.
(3) Studies conducted in adults (aged ⩾19 years) with depressive disorder.

### Exclusion criteria

(1) Studies that adapted the PT only to facilitate access (e.g. home delivery of PT) rather than to address issues of broader cultural relevance.
(2) Studies that used a new PT that was developed specifically for the ethnic group and was not an adaptation of an existing PT.

### Data collation and extraction

The titles and abstracts of each citation identified from our search were inspected independently by two reviewers (N.C. and A.N.) with reference to the inclusion and exclusion criteria and to identify duplicates. The potentially relevant full-text papers were accessed and reviewed independently by the two reviewers. Any disagreements were resolved by consensus and, when this could not be reached, a third reviewer (V.P.) adjudicated. To address the first and second questions of the review, papers that referenced previous publications describing the adaptation process and details of adaptations were also retrieved. Corresponding authors of all papers were contacted to retrieve any additional information regarding these two questions. The questionnaire asked authors to verify the accuracy of information extracted and to add any information regarding process and adaptation that were missing in the papers. Authors were also asked to describe aspects of the PTs that did not require adaptation. Data were summarized in a table based on the theoretical frameworks described below. The quality of included studies was assessed on the following criteria: method of randomization, allocation concealment, blinding of outcome assessment and attrition bias.

### Data analysis

Thematic analysis was used to evaluate the process and nature of the adaptations. Analysis was deductive at first, consisting of predetermined themes applied to data. These themes were based on two frameworks:

(1) The Medical Research Council (MRC) framework for the development and evaluation of complex interventions was used to draw out common elements in the process of cultural adaptation (Craig *et al.* 2008). This framework recommends a phased development process consisting of: modeling/theoretical development, formative work, piloting and evaluation. We categorized the process of adaptation described in the studies into these steps.
(2) Several frameworks were considered for analysis of the nature of the cultural adaptations (Lau, 2006; Hwang, 2009; Castro *et al.* 2010) and the framework of Bernal & Saez-Sanriago (2006) was selected. This framework includes eight dimensions that can be the targets of cultural adaptations: (1) language of the intervention, (2) therapist matching, (3) cultural symbols and sayings (metaphors), (4) cultural knowledge or content, (5) treatment conceptualization, (6) treatment goals, (7) treatment methods, and (8) consideration of treatment context.

Subsequent analysis was inductive and focused on the generation of new categories for the details of the cultural adaptations that emerged from the data.

Meta-analysis was performed using Review Manager version 5.1. The included studies assessed depression (primary outcome) using different psychometric scales; therefore, standardized mean differences (SMDs) were used as appropriate.

The *I*^2^ test was used to measure statistical heterogeneity across studies. A random-effects model was used for the meta-analyses because substantial heterogeneity was observed (*I*^2^ = 90%) (Higgins *et al.* 2003). The uncertainty around heterogeneity was explored with subgroup analyses. A funnel plot was charted to check for publication bias (Egger *et al.* 1997); the plot was asymmetrical suggesting publication bias.

## Results

### Study characteristics

**Fig. 1.**
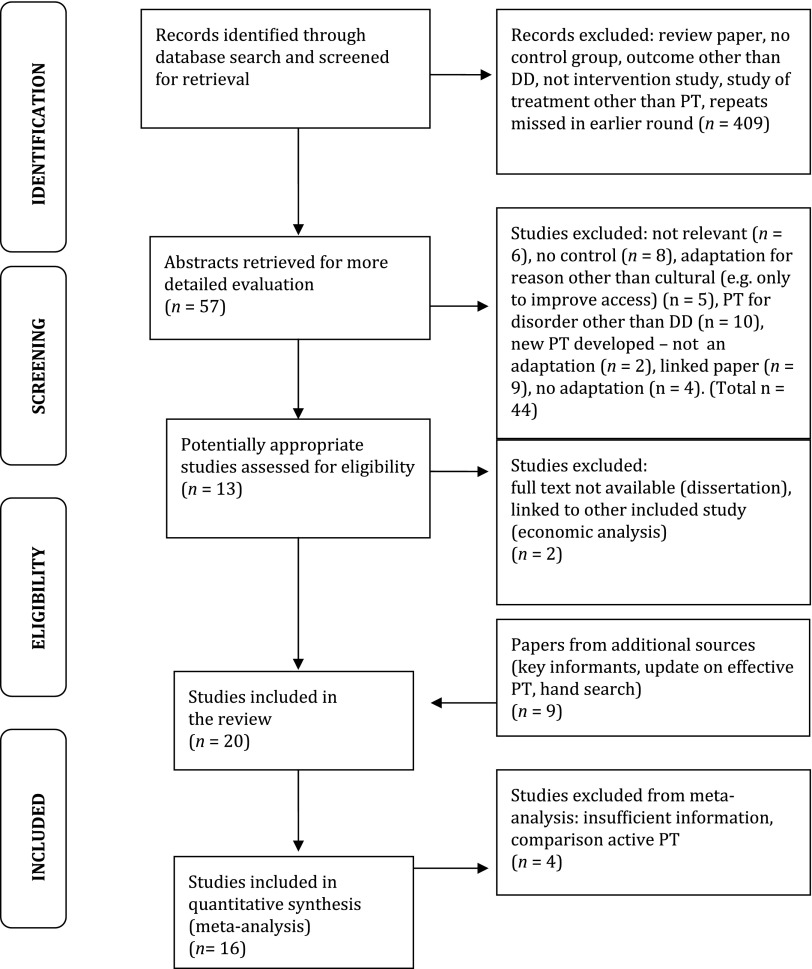
Flow chart of studies included in the review. PT, Psychological treatment; DD, depressive disorder.

After removing duplicates, the electronic search identified 466 potential studies. Figure 1 shows the flow chart of studies from this starting point. Nineteen published studies (Comas-Diaz, 1981; Dai *et al.* 1999; Kohn *et al.* 2002; Araya *et al.* 2003; Bolton *et al.* 2003; Miranda *et al.* 2003*b*; Patel *et al.* 2003; Rojas *et al.* 2007; Rahman *et al.* 2008; Wong, 2008*b*; Grote *et al.* 2009; Hamdan-Mansour *et al.* 2009; Afuwape *et al.* 2010; Beeber *et al.* 2010; Ell *et al.* 2010; Gater *et al.* 2010; Dwight-Johnson *et al.* 2011; Naeem *et al.* 2011; Patel *et al.* 2011) and one unpublished Ph.D. dissertation (Crespo, 2006) were included in the review. The characteristics of the included studies, all of the which were written in English language, are described in Table 1. Information about the process and nature of the adaptations was obtained from the trial papers, their linked papers (*n* = 9) (Andrew *et al.* 2000; Miranda *et al.* 2003*a*; Verdeli *et al.* 2003; Beeber *et al.* 2007; Rahman, 2007; Chatterjee *et al.* 2008; Grote *et al.* 2008; Wong, 2008*a*; Chaudhry *et al.* 2009) and the questionnaires completed by authors (*n* = 10).

**Table 1.** Characteristics of studies included in the systematic review of cultural adaptations of psychological treatments (PTs) for depressive disorder

Author	Country	Class of psychotherapy	Treatment details: modality (individual/group), no. of sessions, frequency, duration	Treatment setting	Therapist qualification and training	Population	Comparison group	Study design	Outcome measure	Result
Afuwape^a^ 2010	UK	CBT-based stepped care package	Individual format	Community	Community health-workers who were psychology graduates with a minimum of 2 months training	Sample size: IG = 16, CG = 16	WL with information on local mental health services	RCT	GHQ-28 at 3 months	Adjusted mean difference 7.76, 95% CI 0.86–14.65, *p* = 0.03
						Black				
						Mean age: IG = 32.8 (10.7), CG = 42.7 (8.4)				
						Female: 60% IG, 75% CG				
Araya^a^ 2003	Chile	Psychoeducation in a stepped care program	Group format; seven weekly sessions and two booster sessions at weeks 9 and 12	Clinic	Social workers and nurses with 12 h of training	Sample size: IG = 104, CG = 109	Usual care including antidepressants or outside referral	RCT	HAMD at 3 and 6 months	Adjusted mean difference −8.89, 95% CI −11.15 to −6.76, *p* < 0.0001
						Female: 100%				
						Mean age:IG = 43.0 (12.8), CG = 42.1 (14.3)				
Beeber^a^ 2010	USA	IPT	Individual format plus mother–child interaction guidance (dyadic); 11 in-home sessions interspersed with five short booster visits	Home	English-speaking master's-prepared psychiatric nurses and project-trained Spanish language interpreters majority who were EHS home visitors	Sample size: IG = 39; CG = 41	Usual care: regular visits by EHS home visitors	RCT	CES-D at 14, 22 and 26 weeks	The mean differences (with s.e.):
						Ethnicity: Latina				Time 2 [−6.8 (2.9) points, *p* = 0.02], Time 3 [−10.6 (3.3) points, *p* < 0.01], Time 4 [−8.4 (3.6) points, *p* = 0.02]
						Female: 100%				
						Mean age: IG = 26.2 (6.1), CG 26.5 (5.8)				
Bolton^a^ 2003	Uganda	IPT	Group format; weekly, 90-min sessions, for 16 weeks	Community	Lay person with 2 weeks training	Sample size: IG = 139, CG = 145 Mean age: IG = 46.4 (16.1); CG = 44.1 (16.5) Female: IG = 50%; CG = 52%	No intervention	Cluster RCT	HCL at 2 weeks	Mean reduction in depression severity: IG: 17.47 (s.e. = 1.1) points; CG 3.55 points (s.e. = 1.1) (*p* < 0.001).
Comas-Diaz 1981	USA	Cognitive therapy	Group format; five sessions over 4 weeks	Clinic	Specialist (doctoral student)	Sample size: IG 8, CG 10 Female: 100% Puerto Rican	Two controls: WL and active PT (BT)	RCT	HAMD at 4 weeks	Change in mean score: IG (CT): 11.46, BT: 10.25, WL: 1.35. Active PT *v.* WL: *F* = 17.564, *p* < 0.001
Crespo 2006	USA	Dynamically orientated art group therapy	Group format; six sessions, 6 weeks	Clinic	Psychologist (doctoral student)	Sample size: 36 Female: 100% Ethnicity: Latina	Two active PT groups: dynamic group therapy and dynamic art group therapy without cultural adaptation	RCT	BDI at 6 weeks	Mean difference 8.4, s.e. = 3.1, *p* = 0.028
Dai 1999	USA	Psychoeducational, CBT	Eight weekly classes followed by discussion group	Community	Psychiatrists	Sample size: IG = 23, CG = 7 Ethnicity: Chinese American Mean age: IG = 71.9 (11.9), CG = 75.6 (7.5) Female: IG = 69.6%, CG = 28.5%	No intervention (waitlist)	Non-RCT	HAMD at 8 weeks	The intervention group had greater improvement in depression score over time than control group. *p* = 0.01 No details available
Dwight-Johnson^a^ 2011	USA	CBT	Eight sessions	Home, community/telephone	Specialists (Social Work) and Social Work students in training	Sample size: IG = 50, CG = 51 Ethnicity: Latina Mean age: IG = 41.1 (9.6), CG = 38.5 (1.2) Female: 78%	Enhanced usual care: providers informed of diagnosis and could provide medications or referrals to outside services	RCT	PHQ at 3 weeks, 3 months and 6 months	Six-month follow-up: *t* = − 2.49, df = 221, *p* = 0.013
Ell^a^ 2010	USA	PST as part of collaborative care	8–12 PST sessions (plus booster sessions if indicated and a PST open-ended patient support group available up to 12 months post-treatment)	Clinic	Graduate social work DDCSs	Sample size: IG = 193, CG = 194. 96% low-income Hispanic Female: IG = 94.8%, CG = 97.4%	Enhanced usual care: education pamphlets, physicians informed of diagnosis, could prescribe antidepressants or refer to community care	RCT	SCL-20 at 6, 12 and 18 months	At 6, 12 and 18 months, adjusted OR 2.46–2.57, *p* < 0.001). IG patients were significantly more likely to have a ⩾50% reduction in depression score and significantly greater odds of depression remission
Gater^a^ 2010	UK	Psychoeducation	Group format; weekly sessions over 10 weeks	Community	Multilingual graduate women trained and supervised by specialists	Sample size: IG = 39, CG (antidepressant) = 42; CG (combined) = 32 Ethnicity: British Pakistani Female: 100%	Two groups: (1) antidepressant; (2) combined treatment (i.e. antidepressant plus psychoeducation)	Cluster RCT	HAMD at 3 and 6 months	IG *v.* antidepressant group: no effect. At 3 months: ES = − 3.3, 95% CI −8.2 to 1.5, *p* = 0.14 At 6 months: ES = − 0.9, 95% CI −4.7 to 3.0, *p* = 0.59
Grote^a^ 2009	USA	IPT	Individual format; one engagement session, eight acute IPT-B sessions, biweekly/monthly maintenance IPT sessions up to 6 months postpartum	Face to face in clinic plus phone	A doctoral level and a master's-level clinician who had supervised training and experience in IPT	Sample size: IG = 25, CG = 28 Pregnant women 18 years old Ethnicity: majority African American (*n* = 33, 62%) Mean age: IG = 24.3 (5.3), CG = 24.7 (5.6)	Enhanced usual care: educational material, referral to community agencies, frequent assessments	RCT	BDI at 3 and 6 months	3 months: *χ*^2^ = 14.02, df = 1, *p* < 0.001; Cohen's *h* = 1.08. 6 months postpartum: *χ*^2^ = 21.16, df = 1, *p* < 0.001; Cohen's *h* = 1.17
Hamdan-Mansour^a^ 2009	Jordan	CBT	Group format; 10 weekly session	Mental health laboratories	Master's-level nurses with experience in psychiatric and mental health nursing received three training sessions	Sample size: IG = 41, CG = 40 Jordanian university students, 55% male, 45% female	No intervention	RCT	BDI at 10 weeks	Difference in score at 10 weeks follow up: IG 12.3 points; CG 5.7 points (*F* = 86.4, *p* < 0.001)
Kohn 2002 [34]	USA	CBT	Group format; 16 weekly	Clinic	Specialist (not specified)	Sample size: IG = 8, CG = 10 African American women Mean age 47 years	Active PT: non-adapted CBT (group intervention)	Non-RCT	BDI at 16 weeks	Decrease in scores at 16 weeks follow-up: IG 12.6 points; CG 5.9 points (*p* value not reported)
Miranda^a^ 2003*b*	USA	CBT	Individual or group format; eight weekly sessions	Clinic	Experienced psychotherapists supervised by licensed clinical psychologist with CBT expertise	Sample size: IG = 90, CG = 89 Female: 100% Mean age: IG = 29.8 (7.9), CG = 29.5 (9.1) African-American (*n* = 117, 43.8%), Caucasian (*n* = 16, 6.0%), Latina (*n* = 134, 50.2%)	Two control groups: enhanced usual care; medication	RCT	HAMD at 3 and 6 months	At 6 months: adjusted mean IG: 7.2; 95% CI 5.0–9.3 and in CG: 10.1; 95% CI 8.0–12.3 (*p* < 0.006)
Naeem^a^ 2011	Pakistan	CBT	Individual format; nine sessions, six twice-weekly and then once a week	Clinic	A psychiatrist and two psychology graduates who received extensive training and ongoing supervision	Sample size: IG = 17, CG = 17 Mean age: IG = 32.3 (8.9), CG = 33.6 (1.0) Female: IG = 82%, CG = 65%	Antidepressants	RCT	HADS at 3 months	Mean difference at 3 months follow-up: 3.8, 95% CI 1.8–5.8, *p* < 0.001
Patel^a^ 2011	India	IPT as part of collaborative stepped care program	Individual session; three psychoeducation sessions merged with up to eight sessions of IPT at weekly/fortnightly intervals	Clinic	Women graduates in any field with no health background	Sample size: IG = 685, CG = 701 Female: 82% Mean age: 46.3 (13.3)	Enhanced usual care: doctor provided with diagnosis and treatment guidelines	Cluster RCT	CIS-R at 2, 6 and 12 months	At 12 months: 30% decrease in the prevalence of CMD among baseline ICD-10 cases (RR 0.70, 95% CI 0.53–0.92); and among the subgroup of patients with depression (RR 0.76, 95% CI 0.59–0.98)
Patel^a^ 2003	India	PST	Individual format, weekly initially, then fortnightly up to six sessions. Course of treatment 3 months	Clinic	Psychologists	Sample size: IG = 150, CG = 150 Mean age: 48.6 Female: 81% both groups	Placebo	RCT	CIS-R at 2, 6 and 12 months	No effect. the mean difference in CISR scores PST *v.* placebo at 2 months: 0.21 (−2.03 to 2.46, *p* = 0.86, ES 0.005) 2–12 months: 0.71 (−1.24 to 2.66)
Rahman^a^ 2008	Pakistan	CBT	Individual sessions; weekly for 4 weeks in the last month of pregnancy, three sessions in the first postnatal month, followed by nine 1-monthly sessions	Home	LHWs, mostly high-school completers, 2-day training with 1-day refresher after 4 months	Sample size: IG = 412, CG = 386 Married women, perinatal depression Mean age: IG = 26.5 (5.2), CG = 27.0 (5.4)	Enhanced usual care: routine visits by LHW	Cluster RCT	HAMD at 6 and 12 months	Mean difference at 6 months −5.86; 95% CI −7.92 to −3.80, *p* < 0.0001
Rojas^a^ 2007	Chile	Psychoeducation in a stepped care program	Group format; eight weekly sessions	Clinic	Midwives and nurses who received 8 h of training and supervision every week	Sample size: IG = 114, CG = 116 Female: 100%, postpartum Mean age: IG = 26.7 (6.4), CG = 26.6 (7.4)	Usual care including antidepressants, brief PT or outside referral	RCT	EPDS at 3 and 6 months	Adjusted mean difference 3 months: −4.5 (95% CI −6.3 to −2.7), *p* < 0.0001 6 months: −2.3 (95% CI −0.50 to 0.04)
Wong^a^ 2008	Hong Kong	CBT	Group format; 10 sessions	Clinic	Mental health specialists	Sample size; IG = 48, CG = 40 Mean age: 37.4 (9.4) Male: 22%	No intervention	RCT	BDI at 10 weeks	Decrease in score at 10 weeks follow-up: IG 9.7; CG 2.6 (*F* = 15.05, *p* < 0.001)

BDI, Beck Depression Inventory; BT, behavior therapy; CBT, cognitive behavior therapy; CES-D, Centre for Epidemiological Studies for Depression Scale; CG, control group; CI, confidence interval; CIS-R, Revised Clinical Interview Schedule; DDCS, diabetes depression clinical specialist; EHS, Early Head Start; EPDS, Edinburgh Postnatal Depression Scale; GHQ-28, 28-item General Health Questionnaire; HAMD, Hamilton Depression Rating Scale; HCL, Hopkin's Symptom Checklist; IG, intervention group; IPT, interpersonal psychotherapy; IPT-B, Interpersonal Psychotherapy - brief; LHW, lady health worker; OR, odds ratio; PHQ, Primary Health Questionnaire; PST, problem-solving therapy; RCT, randomized controlled trial; RR, relative risk; SCL-20, Symptom Checklist 20; s.d., standard deviation; s.e., standard error; WL, waitlist.

Mean age (s.d.) in years.

a Studies included in the meta-analysis.

Out of 20 included studies, four were cluster RCTs (Bolton *et al.* 2003; Rahman *et al.* 2008; Gater *et al.* 2010; Patel *et al.* 2011), 14 individually RCTs (Comas-Diaz, 1981; Araya *et al.* 2003; Miranda *et al.* 2003*b*; Patel *et al.* 2003; Crespo, 2006; Rojas *et al.* 2007; Wong, 2008*b*; Grote *et al.* 2009; Hamdan-Mansour *et al.* 2009; Afuwape *et al.* 2010; Beeber *et al.* 2010; Ell *et al.* 2010; Dwight-Johnson *et al.* 2011; Naeem *et al.* 2011) and two non-RCTs (Dai *et al.* 1999; Kohn *et al.* 2002). Risk of bias of included studies is presented in Appendix 2 online.

### Process of cultural adaptation

The application of the MRC framework to the selected studies is shown in Table 2. Six studies (32%) systematically followed all four stages of the MRC framework in their adaptation process (Bolton *et al.* 2003; Rahman *et al.* 2008; Beeber *et al.* 2010; Ell *et al.* 2010; Gater *et al.* 2010; Patel *et al.* 2011). The remaining studies either did not describe the adaptation process in sufficient detail or omitted some of the stages in treatment development.

**Table 2.** Process of cultural adaptation of psychological treatment (PT) for depressive disorders, based on Medical Research Council (MRC) framework^a^

Author	Modeling/theoretical development	Formative research	Piloting	Evaluation
Araya 2003, Rojas 2007		Writing of manual and asking people to read and comment. Manual used and adapted information from other manuals	No	RCT
Beeber 2010	Literature review, past studies by the PI, FGDs with mothers in EHS (*n* = 4), descriptive survey data from mothers	IDIs and FGDs with EHS English-speaking mothers (*n* = 16), Spanish-speaking mothers (*n* = 57) with severe depressive symptoms; data collected on accessibility (hence, in-home model), stigma (made changes in the medical model focus of IPT), literacy (integrated visual materials), vernacular terms [substituted mothers' terms for depression and disputes, etc. (IPT-specific terms)]	Sixteen non-Hispanic mothers randomized to two groups: intervention and waitlist. Quantitative and qualitative data collected	RCT
Bolton 2003	Multiple consultations with qualitative assessment team and trainee group leaders	Development of working IPT manual draft followed by iterative development and refinement of manual during 10-day lay therapist training	Piloting for manual refinement and treatment adherence/competence during clinician training	Cluster RCT
Ell 2010	Evidence-based practice guidelines and contextual adaptations to the public sector organizational practice setting	Qualitative study of 19 patients that included focus groups and individual structured interviews with respect to knowledge, attitudes and beliefs regarding depression	Pilot study with similar population conducted with cancer patients (*n* = 472). Quantitative data analyzed	RCT
Gater 2010	Consultation with mental health professionals of Pakistani family origin and local voluntary groups	Consultation with Pakistani community centre and the patients. All details regarding form and content of sessions were documented in a manual for future use	Eighteen persistently depressed women enrolled of whom nine women attended at least six of the 10 sessions. Mental health measures were carried out at baseline and on completion of PT. No qualitative results described	RCT
Grote 2009	Clinical observations and literature review		Case series with 12 racially and ethnically diverse women on low incomes	RCT
Kohn 2002	Literature review including published descriptions of treatment approaches used with African American women. Consultation with therapists who have experience treating African American women		Non-randomized study: outcomes of African American women treated in the culturally adapted group (*n* = 8) were compared to African American women treated in the non-adapted group (*n* = 10). Women in the adapted group exhibited a larger drop in average BDI scores	Pilot evaluation
Miranda 2003	Clinical judgment and personal experience used in developing the intervention			RCT
Naeem 2011	Field observations and clinical experience	IDIs with clinical psychologists (*n* = 6) and depressed patients (*n* = 9). FGD with university students (*n* = 34)	Combination of CBT and antidepressants with antidepressants alone (treatment as usual) in 17 primary care clinics. Patients receiving CBT showed statistically significant improvement in depression (*p* < 0.001), anxiety (*p* < 0.001) and somatic symptoms (*p* < 0.000)	Pilot study described
Patel 2003	Literature review. Consultation with experts		Case series with 41 patients. Quantitative and qualitative methods used to determine, compliance, change in morbidity and acceptability of treatment	RCT
Patel 2011	Review of published trials in LMICs. Fourteen consultation meetings with 145 doctors from the Directorate of Health Services, private practitioners and primary health-care staff. Meeting of national and international experts	Case series in four primary health care centers and four private general practice facilities. Mixed methods evaluation with quantitative process indicators and qualitative data (IDIs with key stakeholders) Respondents (total = 89) were: doctors (*n* = 10), patients (*n* = 50), clinic staff (*n* = 17), intervention team (*n* = 12)	Case series in four primary health-care centers and two GP clinics. Mixed methods evaluation with quantitative process indicators and qualitative data (IDIs with 77 patients)	RCT
Rahman 2008	Data synthesis by systematic triangulation of findings from multiple methods, data sources and theories. Review of the synthesized data by panel of experts	FGD with 24 LHW	Case series (*n* = 164) with 42 LHW delivering intervention. Quantitative evaluation with LHWs and mothers	Cluster RCT
		IDIs with six primary care staff including two primary care doctors, two midwives and two traditional birth attendants		
		Data from another epidemiological study by the same author examined for psychosocial risk factors for pre- and postnatal depression		

BDI, Beck Depression Inventory; CBT, cognitive behavior therapy; EHS, Early Head Start; FGD, focus group discussion; GP, general practitioner; IDI, in-depth interview; LHW, lady health worker; LMIC, low- and middle-income country; PI, principal investigator; RCT, randomized controlled trial.

a All studies were not included in this table because they did not describe the process of PT adaptation.

Most authors described the need for cultural relevance to increase acceptability of the PT as the primary rationale for carrying out the adaptation. Other reasons were to address practical barriers to care, such as limited literacy and lack of trained mental health providers, to integrate the PT within existing health services and to improve treatment practices and ensure efficient use of available human resources. Eleven studies (58%) reported the use of the modeling stage (Kohn *et al.* 2002; Bolton *et al.* 2003; Miranda *et al.* 2003*b*; Patel *et al.* 2003, 2011; Rahman *et al.* 2008; Grote *et al.* 2009; Beeber *et al.* 2010; Ell *et al.* 2010; Gater *et al.* 2010; Naeem *et al.* 2011). This involved the selection of a theory-based class of PT, such as cognitive behavior therapy (CBT), mainly through empirical evidence gathered from literature reviews and also through consultations with local stakeholders, for example mental health specialists, primary care doctors and community workers.

The formative research phase using mixed methods to guide the process of development of the preliminary PT manual was reported in eight (42%) studies (Araya *et al.* 2003; Bolton *et al.* 2003; Rahman *et al.* 2008; Beeber *et al.* 2010; Ell *et al.* 2010; Gater *et al.* 2010; Naeem *et al.* 2011; Patel *et al.* 2011). This consisted mainly of collection of qualitative data through focus group discussions and in-depth interviews with the target group (i.e. patients with depressive disorder) and specialist and non-specialist health providers with experience of working with the specific cultural groups or belonging to the same culture (hence having knowledge of the customs/beliefs of that culture). These data were used to refine the treatment's acceptability and feasibility.

Piloting, leading to further refinement of the treatment, was reported in 10 (53%) studies (Kohn *et al.* 2002; Bolton *et al.* 2003; Patel *et al.* 2003, 2011; Rahman *et al.* 2008; Grote *et al.* 2009; Beeber *et al.* 2010; Ell *et al.* 2010; Gater *et al.* 2010; Naeem *et al.* 2011). This involved training the health-care providers to deliver the preliminary version of the treatment and the collection of quantitative and qualitative data either in open trials or in controlled trials with small numbers of patients to improve the acceptability and feasibility of the treatment. Pilot studies also provided an initial assessment of the ability of the treatment to change depression outcomes.

The evaluation phase comprised a controlled trial of the PT comparing the adapted treatment to a control condition in RCTs or non-RCTs, the results of which are described in the following sections.

### Cultural adaptations of PT

The types of PT adapted consisted of CBT (*n* = 10), interpersonal therapy (IPT; *n* = 4), psychoeducation (*n* = 3), problem-solving therapy (*n* = 2) and dynamically oriented therapy (*n* = 1). Details of the adaptations made to the PT, according to type of PT, based on the framework of Bernal & Saez-Sanriago (2006) are presented in Appendix 3 online. Ten authors completed the questionnaire sent to them. All the studies reported adherence to the basic framework and core principles (e.g. the four IPT problem domains) of the original PT so as to preserve fidelity to the treatment. In addition, the authors identified several aspects of the PT that did not require adaptation. These included the phases in which the treatment was delivered, the use of problem-solving techniques and the empathic nature and other ‘non-specific’ aspects of the therapeutic relationship.

Adaptations for language were found in 15 (75%) studies (Comas-Diaz, 1981; Kohn *et al.* 2002; Araya *et al.* 2003; Bolton *et al.* 2003; Miranda *et al.* 2003*b*; Rahman *et al.* 2008; Wong, 2008*b*; Grote *et al.* 2009; Afuwape *et al.* 2010; Beeber *et al.* 2010; Ell *et al.* 2010; Gater *et al.* 2010; Dwight-Johnson *et al.* 2011; Naeem *et al.* 2011; Patel *et al.* 2011) and these went beyond the literal translation to incorporate the use of colloquial expressions to replace technical terms (e.g. renaming ‘homework’ as ‘therapeutic exercise’) and the use of conceptually equivalent idioms of depression (e.g. ‘y'okwetchawa’ and ‘okwekubaziga’ in rural Uganda).

Therapist adaptations, found in 14 (70%) studies (Comas-Diaz, 1981; Araya *et al.* 2003; Bolton *et al.* 2003; Miranda *et al.* 2003*b*; Patel *et al.* 2003, 2011; Rahman *et al.* 2008; Grote *et al.* 2009; Afuwape *et al.* 2010; Beeber *et al.* 2010; Ell *et al.* 2010; Gater *et al.* 2010; Dwight-Johnson *et al.* 2011; Naeem *et al.* 2011), focused on therapist–patient matching to ensure the acceptability and credibility of the therapist by emphasizing shared experiences and awareness of local customs. Some studies reported specific training of therapists in cultural competence to enhance patient engagement. Additionally, cultural factors were considered in the therapist–patient relationship; for example, the therapist adopting a less directive style in some studies and the necessity of setting therapeutic boundaries in others.

The use of metaphors to increase cultural relevance was reported in six (30%) studies (Crespo, 2006; Rahman *et al.* 2008; Grote *et al.* 2009; Dwight-Johnson *et al.* 2011; Naeem *et al.* 2011; Patel *et al.* 2011). These took the form of using material that was culturally relevant, for example a health calendar to monitor homework, the use of stories and local examples with characters resembling the patient's situation and background, and the use of idioms and symbols such as beads for counting thoughts and a mood ladder for rating mood.

Cultural considerations were integrated into the content of the PT in eight (40%) studies (Kohn *et al.* 2002; Bolton *et al.* 2003; Patel *et al.* 2003; Wong, 2008*b*; Grote *et al.* 2009; Ell *et al.* 2010; Naeem *et al.* 2011; Patel *et al.* 2011). These took the form of addressing stressful circumstances such as interpersonal difficulties and focusing on what was in the patient's control. Furthermore, local remedies and practices were integrated into the treatment; for example, massage and religious therapy and additional modules (such as on spirituality) were added to the PT manual, when needed, to contextualize the treatment and address issues relevant to the cultural group.

Adaptations in the dimension of concepts, described in six (30%) studies (Comas-Diaz, 1981; Bolton *et al.* 2003; Wong, 2008*b*; Grote *et al.* 2009; Beeber *et al.* 2010; Naeem *et al.* 2011), involved the communication of the presenting problem and its constructs in a culturally appropriate manner so that they are understandable by the patient and reduced stigma. In particular, this involved addressing the somatic conceptualization of depression by avoidance of psychiatric labels and presenting the problem as a medical illness rather than ‘madness’.

Goals were the least commonly modified dimension, described in four (20%) studies (Bolton *et al.* 2003; Rahman *et al.* 2008; Grote *et al.* 2009; Naeem *et al.* 2011). Adaptations involved development of client-derived treatment goals that were personally and culturally relevant, such as focusing on the health of the family unit rather than the individual. Goals were also extended beyond depression treatment, for example by enhancing roles of group members into community advocates.

Adaptations to methods were reported in 10 (50%) studies (Araya *et al.* 2003; Bolton *et al.* 2003; Miranda *et al.* 2003*b*; Rahman *et al.* 2008; Wong, 2008*b*; Grote *et al.* 2009; Beeber *et al.* 2010; Dwight-Johnson *et al.* 2011; Naeem *et al.* 2011; Patel *et al.* 2011), and included simplifying the steps of treatment and reducing the focus on tasks requiring literacy such as reading and writing.

Finally, 13 (65%) studies reported adaptations to ensure that the PT fit into the patient's broader social context (Comas-Diaz, 1981; Dai *et al.* 1999; Kohn *et al.* 2002; Bolton *et al.* 2003; Miranda *et al.* 2003*b*; Grote *et al.* 2009; Hamdan-Mansour *et al.* 2009; Beeber *et al.* 2010; Ell *et al.* 2010; Gater *et al.* 2010; Dwight-Johnson *et al.* 2011; Naeem *et al.* 2011; Patel *et al.* 2011). These took the form of adaptations to reduce practical barriers and improve access, enhance feasibility and acceptability; for example, flexibility in scheduling sessions, delivering the treatment in a convenient setting or over the telephone, and inclusion of family members if requested by the patient.

### Effectiveness of adapted PTs

The primary outcome, depressive symptoms, was measured using nine different depression scales, most commonly the Beck Depression Inventory (BDI) (*n* = 5); other scales are shown in Table 1. The duration of the follow-up ranged from 2 weeks to 18 months post-treatment. All but two studies (Patel *et al.* 2003; Gater *et al.* 2010) reported the effectiveness of a culturally adapted intervention in depression (see Table 1).

**Fig. 2.**
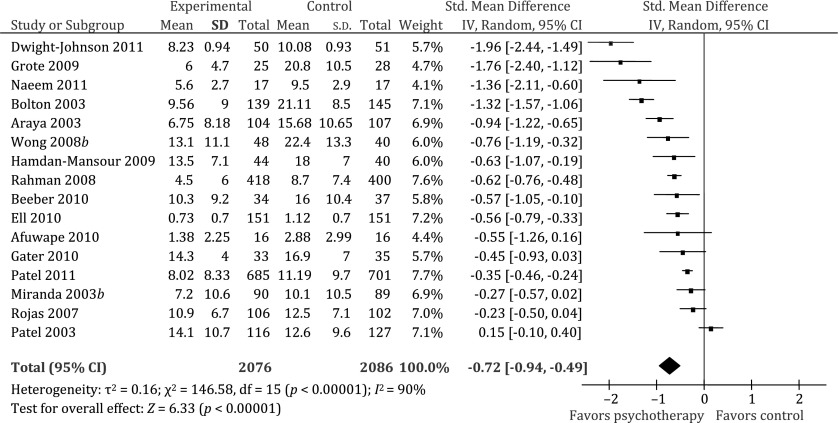
Effect of psychological interventions compared to usual care/no treatment control group. Outcome: depression (higher score indicates greater severity). CI, confidence interval; df, degrees of freedom; s.d., standard deviation.

Sixteen of the 20 studies with 4162 participants were included in the meta-analysis (Bolton *et al.* 2003; Miranda *et al.* 2003*b*; Patel *et al.* 2003, 2011; Rojas *et al.* 2007; Rahman *et al.* 2008; Wong, 2008*b*; Grote *et al.* 2009; Hamdan-Mansour *et al.* 2009; Afuwape *et al.* 2010; Beeber *et al.* 2010; Ell *et al.* 2010; Gater *et al.* 2010; Dwight-Johnson *et al.* 2011; Naeem *et al.* 2011). Four studies were not included in the meta-analysis: three (Comas-Diaz, 1981; Dai *et al.* 1999; Kohn *et al.* 2002) did not provide sufficient information on outcome measures (i.e. they did not report the mean and standard deviation in the two groups, which is necessary to calculate the SMDs) and one (Crespo, 2006) was a poor quality study that administered active (i.e. non-adapted) PT to the comparison group. The pooled weighted SMD showed a statistically significant benefit in favor of the adapted PT over the various control conditions [SMD = −0.72, 95% confidence interval (CI) −0.94 to −0.49], with significant heterogeneity (*χ*^2^ = 146, df = 15, *p* < 0.001, *I*^2^ = 90%) (Fig. 2). This indicates that culturally adapted PTs are efficacious.

Although some of the subgroup analyses revealed differences in effect sizes, none were statistically significant (Table 3). Moreover, significant heterogeneity was observed for each subgroup analysis (*I*^2^ > 50%).

**Table 3.** Subgroup analyses of controlled evaluations of culturally adapted psychological treatment (PT) for depressive disorders

Theme	Subgroup A Effect size (95% CI), *p* value, *I*^2%^	Statistical test for subgroup difference
Participant characteristics		
Female only (*n* = 7)	−0.63 (−0.89 to −0.37), < 0.0 0001, 80	*χ*^2^ = 0.45, df = 1, *p* = 0.50
Male and female (*n* = 9)	−0.72 (−0.94 to −0.49), < 0.0001, 93	
Regional comparison		
Western countries (*n* = 7)	−0.89 (−1.39 to −0.40), 0.0004, 90	*χ*^2^ = 0.83, df = 1, *p* = 0.36
Non-Western countries (*n* = 9)	−0.63 (−0.90 to −0.36), < 0.00 001 92	
Therapist qualification		
Specialist (*n* = 10)	−0.90 (−1.21 to −0.59), < 0.00 001, 83	*χ*^2^ = 3.46, df = 1, *p* = 0.06
Non-specialist (*n* = 6)	−0.40 (−0.87 to 0.15), 0.004, 94	
Format of treatment delivery		
Group format (*n* = 6)	−0.73 (−1.10 to −0.36), 0.0001, 86	*χ*^2^ = 0.11, df = 1, *p* = 0.74
Individual format (*n* = 9)	−0.65 (−0.93 to −0.37), < 0.00 001, 91	
Setting		
Clinical (*n* = 10)	−0.58 (−0.82 to −0.34), < 0.00 001, 86	*χ*^2^ = 1.89, df = 1, *p* = 0.17
Community (*n* = 6	−0.92 (−1.36 to −0.49), < 0.0001, 90	
Type of PT		
CBT (*n* = 6)	−0.88 (−1.29 to −0.48), < 0.0001, 87	
IPT (*n* = 4)	−0.97 (−1.62 to −0.31), 0.004, 95	
Other (social intervention, problem solving) (*n* = 6)	−0.42 (−0.76 to −0.07), 0.02, 86	*χ*^2^ = 3.95, df = 2, *p* = 0.14
Type of comparison group		
Usual care/waitlist (*n* = 7)	−0.80 (−1.20 to −0.40), < 0.0001, 85	*χ*^2^ = 4.79, df = 2, *p* = 0.09
Enhanced usual care (*n* = 5)	−0.58 (−0.82 to −0.34), < 0.00 001, 85	
Medication (*n* = 4)	−0.29 (−0.76 to 0.18), 0.64, 83	
PT delivery		
Pure PT only (*n* = 11)	−0.83 (−1.18 to −0.49), < 0.00 001, 91	*χ*^2^ = 2.31, df = 1, *p* = 0.13
PT as part of package (*n* = 5)	−0.51 (−0.74 to −0.27), < 0.0001, 77	
Allocation concealment		
Adequate (*n* = 11)	−0.56 (−0.79 to −0.33), < 0.00 001, 89	*χ*^2^ = 3.22, df = 1, *p* = 0.07
Unclear/inadequate (*n* = 5)	−1.12 (−1.69 to −0.55), 0.0001, 86	

CBT, Cognitive behavior therapy; IPT, interpersonal therapy; CI, confidence interval; df, degrees of freedom.

Of note, two studies compared the effectiveness of the culturally adapted intervention to the non-adapted intervention directly (Kohn *et al.* 2002; Crespo, 2006). Although both studies reported greater effectiveness of the adapted to the non-adapted PT, we were unable to obtain an integrated effect size because the results did not provide sufficient statistical information to allow this calculation.

## Discussion

In this study we sought to systematically review the literature on adaptations of PTs for depressive disorders for use with ethnic minorities in Western countries and any adaptations of PTs for depressive disorder in non-Western countries. We have used the terms Western/non-Western to reflect the difference between populations in which the psychological interventions were originally developed and tested (always a white European or American population) *versus* populations with different concepts of health and illness.

We identified 20 controlled studies of PTs in these populations. The process of adaptation was reported explicitly in approximately two-thirds of the studies. The common elements within this framework were the selection of a theory-driven class of PT, consultation with a variety of stakeholders in the adaptation process, the use of mixed (qualitative and quantitative) research methods to assess acceptability and feasibility of the PT and pilot studies to evaluate barriers to the delivery of the PT, before the treatment was evaluated in a controlled study (which was an inclusion criterion for the review). Our findings confirm that the procedure used by most investigators is consistent with the methodological framework for the development of complex interventions advocated by the MRC (Craig *et al.* 2008).

Several themes emerged when examining the nature of adaptations to the PT, which were organized using the framework of Bernal & Saez-Sanriago (2006). The use of this framework offers a systematic basis for identifying the various dimensions that need attention in cultural adaptations of PTs. The majority of adaptations were made in the dimensions of language, context and therapist delivering the treatment. Replacing technical terms with colloquial expressions, ensuring therapist–patient matching and cultural competence of therapists were some of the important adaptations reported. This is similar to a previous meta-analysis that reported matching patient to therapist of the same ethnic group in 61% of studies and the same native language (if other than English) in 74% of studies (Griner & Smith, 2006). Other adaptations of salience are the incorporation of local practices into treatment, extending the goal of treatment beyond the patient to include the family, attention to the somatic/physical illness model and simplification of treatment including the use of non-written material. It is important to note that many aspects of the PTs were found to be ‘universally’ applicable, that is they did not require adaptation and the framework and theory of the treatment including treatment phases remained unchanged, respecting the theoretical core of the original PT. Thus, adaptations predominantly reflected efforts to enhance the acceptability of the PTs as opposed to adaptations of core content, thus maintaining fidelity to the original PT.

Despite significant heterogeneity, the meta-analysis of 16 studies confirmed the large effect size of adapted PTs (SMD −0.72). The heterogeneity in our review is not unexpected given the diversity of contexts and PTs in the studies included (Higgins *et al.* 2003). The pooled effect sizes of adapted treatments were similar or greater than those reported in systematic reviews of the non-adapted treatment in ‘Western populations’; for example the effect size of adapted CBT (*n* = 6) (SMD −0.88, 95% CI −1.29 to −0.48) was similar to the effect sizes of non-adapted CBT reported in two reviews [SMD −0.33, 95% CI −0.60 to −0.06 (Peng *et al.* 2009) and SMD −1.34, 95% CI −1.89 to −0.79, *p* < 0.00 001 (Cape *et al*. 2010)] whereas the effect size of adapted IPT (pooled SMD −0.97, 95% CI −1.62 to −0.31) was greater than the non-adapted IPT (SMD −0.11, 95% CI −0.47 to 0.24) (Cape *et al*. 2010). This suggests that adapting PTs for culturally diverse populations can be achieved without compromising treatment effectiveness, and indeed may potentially enhance it.

Of particular interest is the comparative effectiveness of an adapted treatment with a non-adapted treatment in the same population. One previous systematic review (Benish *et al.* 2011) compared adapted PTs with non-adapted PTs (21 studies) and reported a modest effect size (*d* = 0.32) in favor of culturally adapted PTs. That review, however, included studies that were not eligible for our current review because the PTs were for a wide range of psychiatric disorders (e.g. including anxiety disorders, psychoses and behavioral disorders) and across age groups, with 11 out of the 21 (52%) studies being conducted with people aged < 18 years. Although the two comparable studies (Kohn *et al*. 2002; Crespo, 2006) that were eligible for our review also indicate the superiority of an adapted PT compared with the non-adapted version of the same PT in a particular cultural context, both studies had methodological weaknesses and thus this issue deserves further empirical evaluation with better designed studies.

The results of the subgroup analyses found the largest effect sizes in studies of PTs using usual care/waitlist (no treatment) control groups as compared to studies using enhanced usual care controls; and smallest effect sizes in studies using active controls (medication). This is consistent with the findings of other meta-analyses of non-adapted PTs (Peng *et al.* 2009; Cape *et al*. 2010). A considerable part of the heterogeneity in the studies in our review probably stems from the fact that the designation ‘usual care’ can cover a wide range of interventions, ranging from little or none to considerable. Studies that delivered ‘pure PT’ showed larger effect sizes than studies using PT as part of a treatment package. This finding could reflect the fact that the PT may have been less consistently delivered when it was a component of a larger package of care or complex packages may be applied to more complex patient groups where access and delivery of the intervention may pose challenges.

This review is limited by the incompleteness of information for some of the studies. This occurred despite our best efforts to contact authors and search for linked papers. We attempted to look for correlations between the process and degree of adaptation ratings and effectiveness of PT by scoring each study on these dimensions and plotting this against effectiveness. No correlation was found, but this analysis was limited by the relatively small number of studies available. Furthermore, although we have reported studies that have adapted both CBT and IPT, in addition to other PTs, the small number of studies does not allow us to compare the difference in the degree of adaptation across different PTs. More complete information on the process and outcome of cultural adaptations would have been useful for drawing inferences on the method to be adopted for PT adaptation and the likely outcomes. This would have provided a stronger basis for inferring whether some of the processes involved in the adaptation were more important than others. It is also possible that publication bias inflated the estimates of efficacy for the adapted studies (just as it does in the case of non-adapted treatments).

This review extends and updates the literature on PT adaptations by including papers from non-Western countries in addition to those conducted in ethnic minority populations in Western countries, thus offering a global context to the review. Our paper also extends the existing reviews by addressing the process and outcomes of the cultural adaptations of PTs. Our findings demonstrate that cultural adaptations of PTs follow a systematic, and potentially replicable, procedure. Although the diversity of adaptations is a key finding, there were also many aspects of the treatments that did not need adaptation, indicating their universal applicability. Most adaptations were made in the method of delivery rather than in the content of the PT, emphasizing the importance of maintaining fidelity to the original treatment while allowing for greater contextual acceptability. Such adapted PTs show comparable effectiveness for the treatment of depressive disorder as reported in the larger body of evidence evaluating PTs in other populations.

## Supplementary Material

Supplementary Material Supplementary information supplied by authors.

## Supplementary material

For supplementary material accompanying this paper visit http://dx.doi.org/10.1017/S0033291713001785.
