# Supplementary material for: The methods and outcomes of cultural adaptations of psychological treatments for depressive disorders: a systematic review
Source: Psychol Med. 2013 Jul 19;44(6):1131–46. doi: 10.1017/S0033291713001785 (PMC3943384; doi:10.1017/S0033291713001785)
Supplement: Supplementary Material — Supplementary information supplied by authors. [file S0033291713001785sup001.doc]

**SUPPLEMENTARY MATERIAL**

Appendix 1: Search terms used for electronic database search

| **PsycINFO** | **Ovid Medline and EMBASE** |
| --- | --- |
| 1. exp clinical trials/ 2. randomized controlled trials.mp. 3. randomized controlled trial.mp. 4. random allocation.mp. 5. double blind method.mp. 6. clinical trial.mp. 7. placebo$.tw. 8. controlled$.mp. 9. random$.mp. 10. clinical?trial$.mp. 11. compar$.mp. 12. placebo$.mp. 13. doubl$.mp. 14. blind$.mp. 15. 1 or 2 or 3 or 4 or 5 or 6 or 7 or 8 or 9 or 10 or 11 or 12 or 13 or 14 16. exp major depression/ 17. exp "Depression (Emotion)"/ 18. depress$.mp. 19. minor depressi$.mp. 20. 16 or 17 or 18 or 19 21. exp PSYCHOTHERAPY/ 22. psychotherap$.mp. 23. Ethnic Groups/ 24. exp Minority Groups/ 25. exp "Latinos/Latinas"/ or exp Asians/ or exp Blacks/ or exp Mexican Americans/ 26. 23 or 24 or 25 27. 21 or 22 28. 15 and 20 and 26 and 27 | 1. controlled clinical trial.pt. 2. randomized controlled trial.pt. 3. randomized controlled trials/ 4. random allocation/ 5. double blind method/ 6. single blind method/ 7. clinical trial.pt. 8. exp clinical trial/ 9. placebo$.tw. 10. placebos/ 11. random$.tw. 12. research design/ 13. volunteer$.tw. 14. (clin$ adj25 trial$).tw. 15. ((singl$ or doubl$ or trebl$ or tripl$) adj25 (blind$ or mask$)).tw. 16. factorial.tw. 17. cross-over studies/ 18. crossover.tw. 19. Latin square.tw. 20. (balance$ adj2 block$).tw. 21. (animal not human).sh. 22. or/1-20 23. 22 not 21 24. exp Psychotherapy/ 25. psycholog$.tw. 26. or/24-25 27. depression/ 28. exp depressive disorder/ 29. or/27-28 30. exp Adult/ 31. 30 and 29 and 26 and 23 32. exp Ethnic Groups/ 33. exp Minority Groups/ 34. Asian Continental Ancestry Group/ or African Continental Ancestry Group/ or European Continental Ancestry Group/ or African Americans/ or Asian Americans/ 35. exp Hispanic Americans/ 36. latino.mp. 37. 32 or 33 or 34 or 35 or 36 38. 31 and 37 |

Note: 1. For studies from non-western countries, the terms for ethnic minority populations were replaced with individual country names.

2. We used slightly different search terms in PsycINFO data base as many Mesh terms available in Medline and Embase data bases were unavailable in PsycInfo.

Key : [mh] medical subject heading; [sh] subheading; [tw] text words; [pt] publication type; [exp] explode by using this option - MeSH (Medical Subject Headings) terms (as well as any subheading that is the top of a "subheading tree") are "exploded" automatically to retrieve citations that carry the specified MeSH heading (or subheading) and also retrieve citations that carry any of the more specific MeSH headings (or subheadings) indented under the Tree structure; [**$]** dollar sign in Ovid retrieves the word depression as well as the words **depressive, depressed**, and more. For details of the reference to Ovid guide (website:http://ospguides.ovid.com/OSPguides/medline.htm)

Appendix 2: Risk of bias

Afuwape 2010

**+**

Araya 2003

**+**

Beeber 2010

**+**

Bolton 2003

**+**

Comas-Diaz 1981

**–**

Crespo 2006

**–**

Dai 1999

**–**

Dwight-Johnson 2011

**+**

Ell 2010

**+**

Gater 2010

**+**

Grote 2009

**+**

Hamdan-Mansour 2009

**+**

Kohn 2002

**–**

Miranda 2003

**+**

Naeem 2011

**+**

Patel,V 2011

**+**

Patel 2003

**+**

Rahman 2008

**+**

Rojas 2007

**+**

Wong 2008

**–**

**+**

**+**

**?**

**+**

**?**

**–**

**?**

**?**

**+**

**+**

**?**

**?**

**–**

**+**

**+**

**+**

**+**

**+**

**+**

**–**

**–**

**+**

**+**

**–**

**?**

**?**

**?**

**+**

**+**

**+**

**–**

**+**

**?**

**+**

**+**

**+**

**+**

**+**

**+**

**+**

**–**

**+**

**+**

**+**

**?**

**?**

**–**

**+**

**+**

**+**

**+**

**+**

**+**

**+**

**+**

**+**

**+**

**+**

**+**

**+**


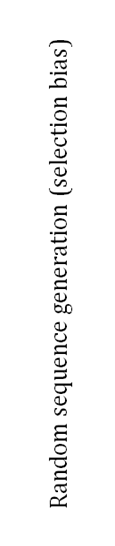

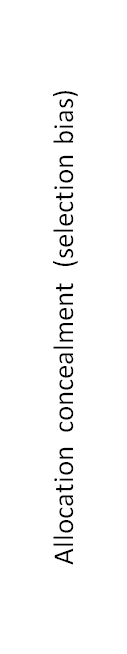

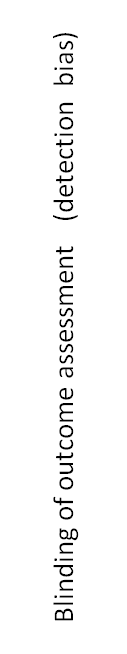

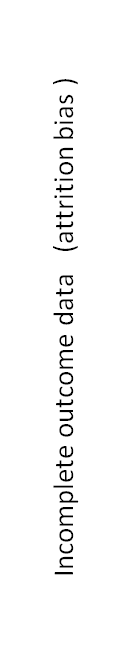


Random sequence generation (selection bias)

Allocation concealment (selection bias)

Blinding of outcome assessment (detection bias)

Incomplete outcome data (attrition bias)

0%

25%

50%

75%

100%

Low risk of bias

Unclear risk of bias

High risk of bias

Appendix 3: Details of cultural adaptations described using Bernal’s framework

| **Adaptation principle** | **Operationalization** | **Rationale** |
| --- | --- | --- |
| **Language** | | |
| **Translation into local language** | Manuals and patient materials were translated into the local language(Afuwape, Craig et al. ; Dwight-Johnson, Aisenberg et al. ; Gater, Waheed et al. ; Comas-Diaz 1981; Araya, Rojas et al. 2003; Bolton, Bass et al. 2003; Miranda, Chung et al. 2003; Rahman, Malik et al. 2008; Ell, Katon et al. 2010; Patel, Weiss et al. 2011) | To match the language spoken by patients and therapists to enhance understanding of the therapy concepts, methods and goals. |
|  | A ‘consecutive, conduit model’ of interpreting was used, in which the interpreter repeated to the (English-speaking) therapist what was said in the source language as exactly as possible in the target language, and relayed as closely as possible the emotional tone and emphasis of the statements used(Beeber, Holditch-Davis et al. 2010). | To ensure therapists could deliver the treatment effectively and follow protocols as accurately as possible |
| **Use of local idioms of depression** | Identification of two highly prevalent local idioms of depression: ‘y’okwetchawa’ and ‘okwekubaziga’(Bolton, Bass et al. 2003) | Local depression syndromes contained symptoms in addition to those described in DSM-IV |
| **Technical terms replaced by colloquial expressions** | All technical terms were translated into colloquial expressions: for example, ‘‘automatic thoughts’’ was renamed‘‘thought traps’’; cognitive distortion of‘‘personalization’’ was rephrased as ‘‘put all the blame and responsibilities onto oneself'; and the term “homework” was modified to “therapeutic exercises.”(Kohn, Oden et al. 2002; Wong 2008; Naeem, Waheed et al. 2010)  Replace the term ‘depression’ with vernacular, culturally congruent terms such as ‘the blues’, ‘sadness’(Beeber, Holditch-Davis et al. 2010)‘stress related illness(Patel, Weiss et al. 2011)  CBT renamed ‘Thinking Healthy’(Rahman, Malik et al. 2008)  Use of terms such as ‘stressed’ or ‘burdened’ or ‘stress related illness’ where necessary and avoidance of psychiatric labels(Rahman, Malik et al. 2008; Grote, Swartz et al. 2009; Beeber, Holditch-Davis et al. 2010; Patel, Weiss et al. 2011). | Using literal translations or translations which are not culturally acceptable is one of the major barriers in therapy  Depression not well understood as a term. Stressed or burden more understandable in the local context. To minimize stigma. |
| **Therapist** | | |
| **Therapist –patient matching** | Therapists ethnically matched and from the same local community, speaking the local language(Afuwape, Craig et al. ; Dwight-Johnson, Aisenberg et al. ; Gater, Waheed et al. ; Comas-Diaz 1981; Araya, Rojas et al. 2003; Bolton, Bass et al. 2003; Miranda, Chung et al. 2003; Rahman, Malik et al. 2008; Ell, Katon et al. 2010; Patel, Weiss et al. 2011) | Local credibility and acceptability, fluency in local dialect, shared experience in norms and events impacting community, and familiarity with local idioms of distress |
|  | Therapist gender matched with that of group(Bolton, Bass et al. 2003) | Respect for local gender values, to promote self-disclosure, |
| **Cultural competence** | Therapists were trained in cultural competence and had considerable experience working with persons of racial-ethnic minority groups(Rahman, Malik et al. 2008; Grote, Swartz et al. 2009; Beeber, Holditch-Davis et al. 2010; Ell, Katon et al. 2010) | To engage with patients and make treatment culturally relevant |
| **Therapist-patient relationship** | Therapist works as a guide adopting a more directive approach(Patel, Chisholm et al. 2003; Naeem, Waheed et al. 2010) | Asian model of "guru" is different from western concept of Socratic dialogue leading to change - patients unable to identify solutions and looked to the counsellor for direction |
|  | Emphasis made on active listening and developing an empathic relationship with the patient(Gater, Waheed et al. ; Bolton, Bass et al. 2003; Rahman, Malik et al. 2008; Patel, Weiss et al. 2011) | To ensure patient engagement in the treatment process |
|  | Therapists trained in setting boundaries within the therapeutic relationship(Beeber, Holditch-Davis et al. 2010) | To structure informal nature of the home setting |
|  | Participants were addressed and greeted in a traditional manner and at the beginning of each session they were reminded about confidentiality(Gater, Waheed et al.) | To make the engagement process more effective. |
| **Use of non mental health workers** | Use of LHW within the primary care system, nurses, community workers(Bolton, Bass et al. 2003; Rahman, Malik et al. 2008; Beeber, Holditch-Davis et al. 2010; Patel, Weiss et al. 2011) | To reduce stigma and preserve patient’s privacy (especially during home visits) from inquisitive neighbours/family members. Also to make best use of already available and/or low cost resources. |
| **Metaphors** | | |
| **Use of material with cultural relevance** | Displaying culturally relevant pictures in the therapist’s office(Grote, Swartz et al. 2009) | To make participant comfortable in treatment setting where other people look like her |
|  | Designation of a “health corner” in each house, and a “health calendar” provided to each mother to monitor homework and chart progress. Using culturally appropriate illustrations, for example, characters depicting mothers and infants(Rahman, Malik et al. 2008) | By using the illustrated characters, the health workers could avoid direct confrontation with women and their families where it was not appropriate. It facilitated work with non-literate women |
| **Use of stories, local examples** | Examples from religious texts (the life of the Prophet Muhammad and Quran were used to clarify issues)(Naeem, Waheed et al. 2010) | Patient could understand new ideas when described using familiar stories/figures and enhanced acceptability of treatment |
|  | The content of examples and vignettes in the treatment workbook were modified to use Latino names and reflect situations relevant to rural Latinos(Dwight-Johnson, Aisenberg et al.) | To increase cultural relevance |
| **Use of idioms/symbols** | Mood ratings were elicited from patients by showing a picture of a mood ladder with each rung depicting a higher or lower level of mood intensity(Patel, Weiss et al. 2011) or by using facial expressions rather than words(Rahman, Malik et al. 2008) | To convert an abstract concept such as mood into a more concrete, easy to understand concept |
|  | Emotional symptoms explained in physical terms with use of analogies e.g. disability caused by depression similar to inability to walk when a leg is fractured(Patel, Weiss et al. 2011). | To reduce perceived stigma of having a mental illness |
|  | Use of beads for counting thoughts(Naeem, Waheed et al. 2010) | Culturally familiar method |
|  | Mural formation, an artistic expression form associated with Latino heritage was integrated into the group therapy(Crespo and B. 2006) | To make the art therapy more relevant |
| **Content** | | |
| **Addressing stressors** | Identifying interpersonal stressors triggering depression and working on breaking social isolation, and building interpersonal skills to handle stressors more effectively(Bolton, Bass et al. 2003) | To demystify depression by providing interpersonal context to understanding depressive symptoms; Mobilize interpersonal antidepressant strategies, eg., break social isolation, generate options, identify advocates who can help |
|  | Emphasized the exploration and modification of dysfunctional rules relating to family and interpersonal relationships(Wong 2008). | Chinese people have many family and interpersonal relationship rules that may become a potential source of stress |
|  | Psychoeducation provided to dispel treatment misconceptions, reduce stigma, and enhance therapeutic alliance with referral services(Ell, Katon et al. 2010; Patel, Weiss et al. 2011). | To facilitate patient-provider communication and financial and social resource access. |
| **Incorporation of local practices into treatment** | Use of local remedies and practices, in addition to the ‘core’ treatment, for example, head and neck massage, meditation, attending religious practices and ceremonies. Therapists were advised to be respectful towards patient’s practices which were culturally acceptable, for example use of traditional medicines, attending faith healers/spiritual healers etc(Kohn, Oden et al. 2002; Patel, Chisholm et al. 2003; Naeem, Waheed et al. 2010; Patel, Weiss et al. 2011). | To contextualise the treatment to address issues that are relevant to the cultural group |
| **Additional therapy modules to address cultural factors** | Culturally specific sections of content added as therapy modules. These include: 1) creating healthy relationships; 2) spirituality; 3) African American family issues, and 4) African American female identity(Kohn, Oden et al. 2002) | To contextualize the therapy manual to address issues relevant to African American women |
|  | Greater focus on teaching patients on identifying thoughts and emotions, since they generally find it difficult to separate thoughts from emotions(Naeem, Waheed et al. 2010) | Explain psychological concepts that may be unfamiliar |
| **Concepts** | | |
| **Somatic** | The bio-psycho-spiritual-social model was used in initial formulation. Similarly, somatic symptoms were focused in initial formulation.i.e. physical symptoms were included in the first thought diary to highlight the importance of the physical symptoms and their association with thoughts and mood(Naeem, Waheed et al. 2010) | To preserve congruence with cultural beliefs and physical/somatic belief models of illness causation |
| **Social** | “Deconstructing” poverty as a series of role transitions (either loss of finances/resources or failure to bring on a desired improvement(Bolton, Bass et al. 2003) | To avoid over attribution of depression to poverty, promote more nuanced discussion of interpersonal and social roles that have been impacted by poverty, and mobilize support systems to assist in its improvement |
|  | Loneliness and social isolation not identified as problem area in IPT(Bolton, Bass et al. 2003) | People were socialised and participated in community activities on a daily basis |
|  | Engagement session case management to address basic needs(Grote, Swartz et al. 2009) | To identify barriers to care, address treatment ambivalence, and increase likelihood of participation in treatment to make treatment culturally relevant to and effective for depressed mothers and expectant mothers on low incomes for whom multiple stressful social problems and depression are closely linked |
|  | Learned helplessness therapy strategies were added to Beck's cognitive therapy(Comas-Diaz 1981) | Person facing overwhelming social difficulties feel powerless resulting in an inability to effectively influence the course of events |
|  | Instead of promoting the ‘sick role’, IPT adapted to respect the value that of African American mothers held of being strong, independent and single mother/primary earner images that they had seen in their mothers(Beeber, Holditch-Davis et al. 2010) | Acknowledgement of local traditions and values |
|  | Providing the "sick role," e.g., assigning of y’okwetchawa’ and okwekubaziga as medical conditions that can be treated; in the meantime patient’s and family’s expectations for optimal functioning need to be reduced temporarily until symptom remission. Patient is encouraged to ask family and community for help and support(Bolton, Bass et al. 2003) | To reduce guilt and burden due to symptoms and functional impairment while mobilizing interpersonal resources |
| **Religious** | Therapists need to understand the cause and effect relationship in Asian culture, which might be based on cultural or religious causes of events. ie causes of illness might be based on a combination of these factors, along with those from old Greek, Chinese and Indian medicine(Wong 2008) | To overcome barriers to recruitment and attendance for the subjects |
| **Goals** | | |
| **Client derived goal** | Focus on mother and infant health rather than maternal depression and have an a priori agenda of achieving optimal infant development through the intervention(Rahman, Malik et al. 2008) | Infant care was seen as a shared responsibility and this helped engage not only the mother, but the whole family in a supportive role for the mother |
| **Clarifying goals** | Emphasis on non-material goals, e.g., improving depression and social functioning, rather than provision of material goods or services(Bolton, Bass et al. 2003) | Clarification of treatment expectations; community accustomed to receiving financial and health assistance from NGOs |
| **Extending goals beyond depression** | Emphasis on educating patient, while respecting their understanding and explanation of their problems, especially addressing stigma and misperceptions(Naeem, Waheed et al. 2010) | Patients generally had no or minimum knowledge of illness and its treatment, especially psychotherapy |
|  | Emphasis was laid on group members' role development into community advocates as depression improves(Rahman, Malik et al. 2008) | Underscoring impact of depression treatment on wider community development goals, e.g., farming initiatives, school attendance, etc |
| **Methods** | | |
| **Structural adaptations** | Delivering treatment by telephone, intensive outreach and shortening treatment with reduced number of sessions(Dwight-Johnson, Aisenberg et al. ; Miranda, Chung et al. 2003; Patel, Weiss et al. 2011) | To increase accessibility and feasibility |
|  | Slowing down the pace of treatment(Miranda, Chung et al. 2003) | To ensure patient understands and is in step with treatment |
| **Adaptation in techniques used to deliver treatment** | Less use of written material and limiting homework to simple suggestions rather than writing tasks(Araya, Rojas et al. 2003; Naeem, Waheed et al. 2010) | To overcome limited literacy levels |
|  | Structured problem solving with the help of illustrations: eg. In each session mothers were shown two sequence of pictures. The first sequence of pictures was used to facilitate their understanding of the problem. The second sequence was used to help them think of as many possible ways of solving the problem as she can. Subsequently picking the option that seemed to offer the most favourable balance and putting it into action(Rahman, Malik et al. 2008) | Community health workers are able to understand and employ these techniques and they are easily understandable by the patient. |
|  | Each session employs the three-step approach that is repeated throughout the programme(Rahman, Malik et al. 2008). | Simplification of the methods by dividing it into simple steps is necessary so that community health workers are able to understand and employ these techniques. |
|  | Explicit treatment protocols with supporting materials; weekly supervision of all therapists; manualized materials used with all mothers within which mothers were given choices and set the pace of the therapy(Beeber, Holditch-Davis et al. 2010) | Fidelity achieved through standardization without sacrificing mothers' autonomy and choice |
|  | Avoidance of culturally incompatible confrontational styles of communication(Bolton, Bass et al. 2003) | To assist the building of interpersonal skills while respecting local codes of power and intimacy |
|  | The group leaders delivered mini-lectures and provided a detailed explanation of the exercises and worksheets to the participants(Wong 2008). | To facilitate the understanding of the cognitive and behavioral processes, and the learning of cognitive and behavioral skills |
|  | Simple written contract was done with each mother; mothers who could not read or write completed a verbal contract(Beeber, Holditch-Davis et al. 2010) | Treating the mother as a full partner relayed an essential message of expected self-efficacy |
|  | Use of handouts: with simplified explanation of thepsychological treatment for patients and familymembers(Patel, Weiss et al. 2011). | To make treatment understandable |
| **Context** | | |
| **Increase accessibility** | Flexibility in scheduling and conducting sessions(Beeber, Holditch-Davis et al. 2010; Patel, Weiss et al. 2011), for example, so patients could attend important village-wide events (e.g., funerals) and provisions made to allow for interruptions during sessions(Bolton, Bass et al. 2003) | Sensitivity to local community customs to maximize acceptability of the  treatment and improve buy-in from wider community and respect the patients autonomy and choice |
|  | Delivering treatment in a convenient, medical setting -- "one-stop-shop"(Miranda, Chung et al. 2003; Grote, Swartz et al. 2009; Hamdan-Mansour, Puskar et al. 2009; Naeem, Waheed et al. 2010; Patel, Weiss et al. 2011) | To increase access to care and reduce participant burden |
| **Ensure feasibility** | Time keeping through prayer calls rather than watches(Naeem, Waheed et al. 2010) | Participants view time of day according to religious rituals |
|  | Provision of free bus passes, childcare services facilitation of access to needed social services(Grote, Swartz et al. 2009) | To reduce practical barriers to care to help meet participant basic needs, eg. food, housing, which have priority over psychological needs |
|  | The groups took place in a culturally acceptable venue with provision of childcare facilities. Food was provided at the end of each session(Gater, Waheed et al.) | To ensure that women could participate in the PT by taking care of their child rearing responsibilities |
|  | Additional phone calls made to remind patients of the time, day and date of sessions(Gater, Waheed et al. ; Hamdan-Mansour, Puskar et al. 2009; Naeem, Waheed et al. 2010; Patel, Weiss et al. 2011) | To ensure adherence |
|  |  |  |
| **Ensure acceptability** | Treatment delivered in single sex groups(Kohn, Oden et al. 2002; Bolton, Bass et al. 2003) | To facilitate disclosure which may be hampered in mixed gender groups |
|  | Disclosure to community about general purpose of groups(Bolton, Bass et al. 2003) | Rigid explanantion of confidentiality could be misconstrued as secrecy or conspiracy |
|  | Participants (women) were collected for group sessions by taxi accompanied by a female transport facilitator/companion(Gater, Waheed et al.) | To ensure that the family and community did not object to women going out alone with a taxi driver |
|  | Patients were given the option to conduct their first session in person rather than on the telephone(Dwight-Johnson, Aisenberg et al.). | In response to the cultural values that emphasize personalismo (i.e. personal rapport) |
|  | The adaptations were done specifically to allow the inclusion and retention of low-income mothers; extensive adaptations to allow for harsh circumstances and stressors as well as single-parent status, acknowledgement of genuine threats (environmental dangers; discrimination) and previous trauma of relocation, migration experiences and current separation from family and other children(Beeber, Holditch-Davis et al. 2010) | Contextual stressors were seen as one of the major contributors to depression |
|  | Inclusion of family members in treatment if preferred by patient(Ell, Katon et al. 2010).  Addition of specific treatment materials for husbands to acknowledge their place in the home(Beeber, Holditch-Davis et al. 2010) | Acknowledges the central role of the family in the treatment process |
|  | Keeping the group closed to facilitate cohesion(Kohn, Oden et al. 2002) | Participants have greater sense of belonging |
|  | Delivery in the form of ‘classes’(Dai, Zhang et al. 1999) | Chinese individuals have high value for education |
|  | In the Latina mothers, male partners and husbands were part of the scene; we showed deference and respect for these heads of households by gaining their understanding and trust of the therapy process before beginning with the mother's treatment(Beeber, Holditch-Davis et al. 2010) | Acknowledgement of the traditions and values allowed the therapy teams entry into these families and increased the possibility of follow-through |
|  | Format of treatment delivery modified depending upon context e.g. individual format preferred in some settings due to concerns about confidentiality (Patel 2011) while group format preferred in another setting as it was congruent with extended family concept(Comas-Diaz 1981) | To suit cultural preference |
